# Mismatch Repair Status and Lymph Node Ratio in Survival Prediction of Stage II/III Rectal Cancer Patients: A Comprehensive Analysis of a Multi‐Center Retrospective Study

**DOI:** 10.1002/cam4.70756

**Published:** 2025-04-02

**Authors:** Kailong Zhao, Wenwen Pang, Xinyu Liu, Kemin Ni, Weifeng Gao, Zhiquan Tan, Jun Xue, Weizheng Liang, Xueliang Wu, Xipeng Zhang, Xiaomin Su, Chunze Zhang

**Affiliations:** ^1^ School of Medicine Nankai University Tianjin China; ^2^ Department of Colorectal Surgery, Tianjin Union Medical Center The First Affiliated Hospital of Nankai University Tianjin China; ^3^ Department of Clinical Laboratory Tianjin Union Medical Center Tianjin China; ^4^ Tianjin Medical University Tianjin China; ^5^ The Institute of Translational Medicine Tianjin Union Medical Center Tianjin China; ^6^ Tianjin Institute of Coloproctology Tianjin China; ^7^ Department of Information Tianjin Union Medical Center Tianjin China; ^8^ Department of General Surgery The First Affiliated Hospital of Hebei North University Zhangjiakou China; ^9^ Institute of Cancer, the First Affiliated Hospital of Hebei North University Zhangjiakou Hebei China; ^10^ Department of Immunology Nankai University School of Medicine, Nankai University Tianjin China

**Keywords:** adjuvant chemotherapy (ACT), lymph node ratio (LNR), microsatellite instability (dMMR), survival

## Abstract

**Background:**

The microsatellite status (dMMR vs. pMMR) in colorectal cancer can serve as a guiding factor for patient prognosis and treatment, where dMMR status indicates a better prognosis and often obviates the need for adjuvant chemotherapy (ACT). Conversely, a higher lymph node ratio (LNR) is associated with a poorer prognosis. This study aims to elucidate the prognostic significance of LNR and MMR status in relation to ACT in stages II and III colorectal cancer.

**Methods:**

A total of 1946 patients who underwent radical resection for colorectal cancer and were pathologically staged as II and III from three medical centers between 2012 and 2019 were selected. Among them, 1104 patients were included after MMR status was tested and postoperative chemotherapy was administered, along with other clinical information. MMR (mismatch repair) status was determined via pathological immunohistochemistry (IHC), and LNR was calculated. Patients were divided into three groups based on the LNR value and subjected to Kaplan–Meier and Cox regression analysis to assess the impact of MMR, LNR, and ACT on overall survival (OS) and disease‐free survival (DFS).

**Results:**

A total of 6.47% of stage II and III colorectal cancers were detected as dMMR. Significant differences in OS and DFS between dMMR and pMMR patients were observed when the LNR ranged from 0.03 to 0.31, with pMMR patients showing a better prognosis. Stratified analysis with ACT revealed that postoperative chemotherapy did not affect the prognosis within the dMMR patient group. However, compared to the pMMR group, dMMR patients experienced significantly adverse effects on prognosis after receiving postoperative chemotherapy (*p* < 0.05). This result was more pronounced in the stratified analysis based on LNR (0.03–0.31) (*p* < 0.01).

**Conclusions:**

Integrating LNR based on the microsatellite status of colorectal tumors provides comprehensive prognostic predictions, enhancing postoperative prognostic considerations for tumor patients. Additionally, our study suggests that patients with stage II and III colorectal cancer with dMMR status do not require any adjuvant chemotherapy postoperatively.

**Abbreviations**
ACT: adjuvant chemotherapy
DFS: disease‐free survival
dMMR: mismatch repair deficiency
ELN: examined lymph nodes
HR: hazard ratio
LNR: lymph node ratio
LVI: lymphovascular invasion
MMR: microsatellite instability
OS: overall survival
PLN: positive lymph nodes
pMMR: protein mismatch repair deficiency
PNI: perineural invasion

## 1 Introduction

Colorectal cancer currently ranks second in cancer‐related mortality worldwide [1, 2], imposing a significant healthcare burden. With the implementation of colorectal cancer screening programs globally, there has been a decreasing trend in the incidence of colorectal cancer in Europe and the United States in recent years [3, 4]. However, in China, the lack of nationwide promotion and accessibility to such screening programs has led to persistently high incidence rates of colorectal cancer [5]. Therefore, it is crucial to identify important prognostic factors or predictive markers for colorectal cancer mortality and prognosis.

In recent years, research has suggested that microsatellite status (MMR) and lymph node ratio (LNR) can serve as relevant prognostic indicators and can guide treatment decisions for colorectal cancer patients. MMR status can be classified into two types: microsatellite instability (dMMR) and microsatellite stability (pMMR). dMMR is defined as the presence of mutations or deletions in one or more mismatch repair genes (MLH1, MSH2, MSH6, or PMS2) [6, 7, 8]. While MLH1 and MLH2 are commonly mutated or deleted genes in Europe and the United States [8, 9], our study found that PMS2 deletion accounted for the highest proportion, which may be influenced by factors such as ethnicity. Studies have indicated that the most prevalent deficiency among mismatch repair proteins in Chinese populations is PMS2, a pattern that is also observed in similar research [10, 11].

Studies on dMMR/MSI patients are relatively scarce, and the number of such individuals is low; hence, the impact of rectal adenocarcinoma treatment remains a matter of debate. Some studies have indicated that dMMR is most commonly observed in patients with stage II and III colorectal cancer [12]. Existing research on dMMR in colorectal cancer generally suggests a favorable prognosis for patients, although there are opposing viewpoints as well [13]. Previous studies have indicated that dMMR/MSI can serve as a prognostic predictor gene [14, 15], and clinical studies have shown that patients with this type of colorectal tumor derive limited benefits from fluorouracil‐based therapy [16, 17]. The necessity of adjuvant chemotherapy (ACT) for patients with stage II and III dMMR colorectal cancer remains a topic of intense debate.

Lymph node metastasis is a significant route of spread in colorectal cancer patients, with a higher number of metastases correlating with poorer prognosis. However, this prediction is contingent upon the extent of lymph node dissection [18, 19]. On the other hand, as research progresses, it has been recommended that the number of lymph nodes examined during colorectal cancer surgery should not be less than 12 [20]. It has been found that the lymph node ratio (LNR), which is the ratio of positive lymph nodes to total lymph nodes, is a better predictor of postoperative survival in colorectal cancer patients [21, 22, 23]. Additionally, the factor of the lymph node ratio (LNR) provides a more comprehensive explanation of the role of lymph nodes. Previous studies have also confirmed that LNR is an independent risk factor for colorectal cancer patients [24], and it is an important risk factor for patient survival after surgery [25]. Furthermore, existing studies suggest that higher LNR values are often associated with poorer prognosis.

Based on these studies, our research aims to explore whether LNR, combined with MMR status, can provide more accurate postoperative prognostic predictions and guidance for patients with stage II and III colorectal cancer, as well as provide recommendations regarding the need for adjuvant chemotherapy for dMMR patients postoperatively.

## 2 Methods

### 2.1 Study Population and Design

This study collected data from patients diagnosed with rectal cancer at three major medical centers (Tianjin Union Medical Center, Tongji Hospital, The Third Central Clinical College of Tianjin Medical University) between 2012 and 2019. The inclusion criteria for patient data were as follows: (1) The inclusion criteria encompassed individuals with stage II rectal adenocarcinoma (age ≥ 18 years, ≤ 80 years); (2) Preoperative chest, abdominal, and pelvic CT or MRI scans; (3) Confirmation of no tumor residue by CT or MRI at the first follow‐up visit; (4) Absence of abnormal bleeding tendencies; (5) Patients who did not receive preoperative neoadjuvant therapy or palliative surgical resection; (6) No severe acute or chronic illnesses in the 3 months preceding the study, including myocardial infarction, stroke, congestive heart failure, gastrointestinal bleeding within 1 year prior to the study, diabetes, and uncontrolled infections within the 3 months preceding the study. Additionally, patient data included admission information (age, gender), pathological information (T stage, N stage, TNM stage, tumor differentiation, postoperative lymph node status, tumor size, vascular invasion (VNI), perineural invasion (PNI), microsatellite status (MMR)), and postoperative treatment information (whether adjuvant chemotherapy (ACT) was administered). This study received approval from the medical ethics committee.

### 2.2 Factor Definitions

In our study, the determination of patients' tumor microsatellite status was conducted using immunohistochemical (IHC) staining analysis of MMR genes/proteins (MLH1, PMS2, MSH6, and MSH2), which were performed by senior pathologists to ensure accuracy. In this study, the presence of one or more deficient MMR proteins was defined as dMMR, otherwise defined as pMMR.

The LNR value (PLN/ELN) was calculated by first dividing patients into two groups: those with a value of 0 and those with a value greater than 0. By employing statistical testing with X‐tile, different values are used as cutoff groups, and the test result with the smallest p‐value is considered the optimal cutoff for the dataset [26, 27]. Subsequently, patients with values greater than 0 were further stratified into two groups using X‐tile software to find the optimal cutoff value for LNR [28]: 0.03–0.31 and 0.30–1.00. Patients with values between 0 and 0.03 were excluded from the discussion as their number was zero. The cutoff value is recorded in Figure 7.

Regarding postoperative adjuvant treatment, treatment plans were determined based on tumor pathology, patient condition, and preference, following the NCCN guidelines for colorectal cancer and discussions by a multidisciplinary medical team. A multidisciplinary team comprising pathologists, oncologists, and surgical physicians evaluates the postoperative pathological condition of the patient, taking into account the patient's clinical status to discuss the optimal mode of medication, duration of use, and timing of postoperative adjuvant therapy. Most patients received postoperative adjuvant chemotherapy, thus patients receiving chemotherapy were grouped together, without further subdivision based on chemotherapy regimen. Specific chemotherapy regimens (including FOLFOX, CAPEOX, FOLFIRI, capecitabine, or fluorouracil) were recorded，Usually, the start time for postoperative adjuvant chemotherapy for patients is 2–4 weeks after surgery, with a medication plan of one cycle every 2 weeks. Depending on the patient's pathology and physical fitness, the duration of postoperative chemotherapy is generally about 6 months. Patients who did not receive chemotherapy were grouped separately.

Differentiation grading (Grade) of tumor was defined as follows: Grades 1 and 2 were classified as Poorly differentiated, while Grades 3 and 4 were classified as Well differentiated. Tumors with unclear differentiation grading were categorized as Special type.

### 2.3 Patient Follow‐Up

All patients were followed up until the last contact or death. Relevant information was obtained from medical records or death certificates. Each follow‐up included clinical information such as CEA levels, liver ultrasound examination, chest, abdominal, and pelvic CT or MRI scans, and colonoscopy when indicated based on clinical status.

### 2.4 Statistical Analysis

Stratified analysis of the factors was conducted using GraphPad Prism 9.5 to analyze survival differences between different groups. Kaplan–Meier survival curves and Log‐rank tests were utilized. COX multivariate regression analysis was performed using IBM SPSS Statistics 26. Data processing was conducted using Rstudio. We utilized the Chi‐squared test and Fisher's exact test for comparing categorical variables, as well as the independent samples *t*‐test and Mann–Whitney *U* test for comparing continuous variables, with statistical significance set at *p* ≤ 0.05.

## 3 Results

### 3.1 Inclusion of Clinical Characteristics

**FIGURE 1:**
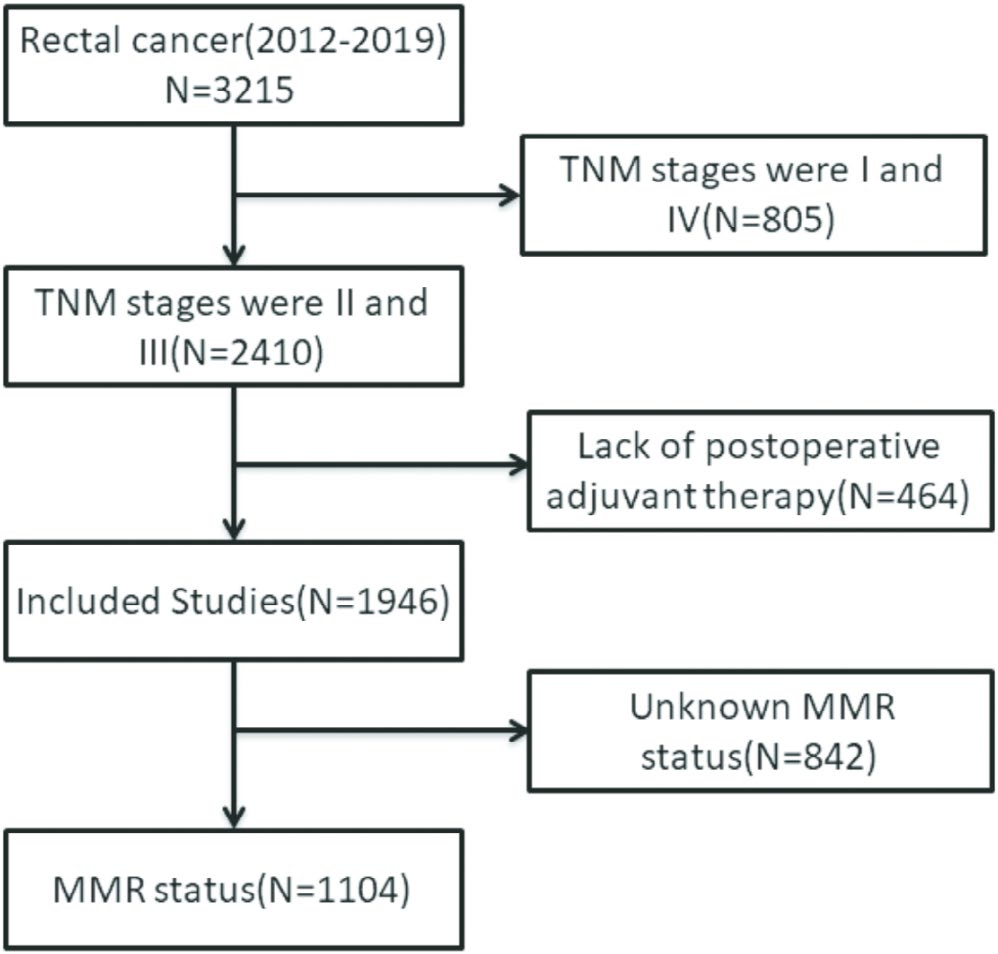
Flowchart of study population selection. MMR, microsatellite instability.

In accordance with the research objectives, we included 1946 patients with pathological TNM stages II and III rectal cancer, among whom 1104 patients had MMR status available, making them the focus of this study (Figure 1). Of these 1946 patients, 1218 (62.59%) were male, in the age group of 50–69; the proportion of individuals is the largest, with 1327 people (68.19%), with an average lymph node ratio (LNR) of 0.20. Patients with dMMR pathology accounted for 6.47% (Table 1). The average follow‐up duration was 58.36 months.

**TABLE 1:** Baseline characteristics of the population.

Variables	Total (%) *N* = 1946
Age	
< 50	202 (10.38%)
50–69	1327 (68.19%)
≥ 70	417 (21.43%)
Sex	
Female	728 (37.41%)
Male	1218 (62.59%)
T stage	
1	11 (0.57%)
2	83 (4.27%)
3	1684 (86.54%)
4	168 (8.63%)
N stage	
0	975 (50.10%)
1	649 (33.35%)
2	322 (16.55%)
LNR (mean = 0.20)	
0	1037 (53.29%)
0.03–0.31	712 (36.59%)
0.32–1.00	197 (10.12%)
TNM	
II	975 (50.10%)
III	971 (49.90%)
Grade	
Well (3/4)	1361 (72.05%)
Poorly (1/2)	396 (20.96%)
Special type	132 (6.99%)
Size (cm)	
< 5	1165 (59.87%)
≥ 5	781 (40.13%)
ACT	
No	583 (29.96%)
Yes	1363 (70.04%)
VNI	
No	1717 (88.23%)
Yes	229 (11.77%)
PNI	
No	1717 (88.23%)
Yes	229 (11.77%)
MMR	
dMMR	126 (6.47%)
pMMR	978 (50.26%)
Unknown	842 (43.27%)

Abbreviations: ACT, adjuvant chemotherapy; dMMR, mismatch repair deficiency; LNR, lymph node ratio; LVI, lymphovascular invasion; MMR, microsatellite instability; pMMR, protein mismatch repair deficiency; PNI, perineural invasion.

### 3.2 Prognostic Analysis of Patients With Stages II and III by Different MMR Status

**FIGURE 2:**
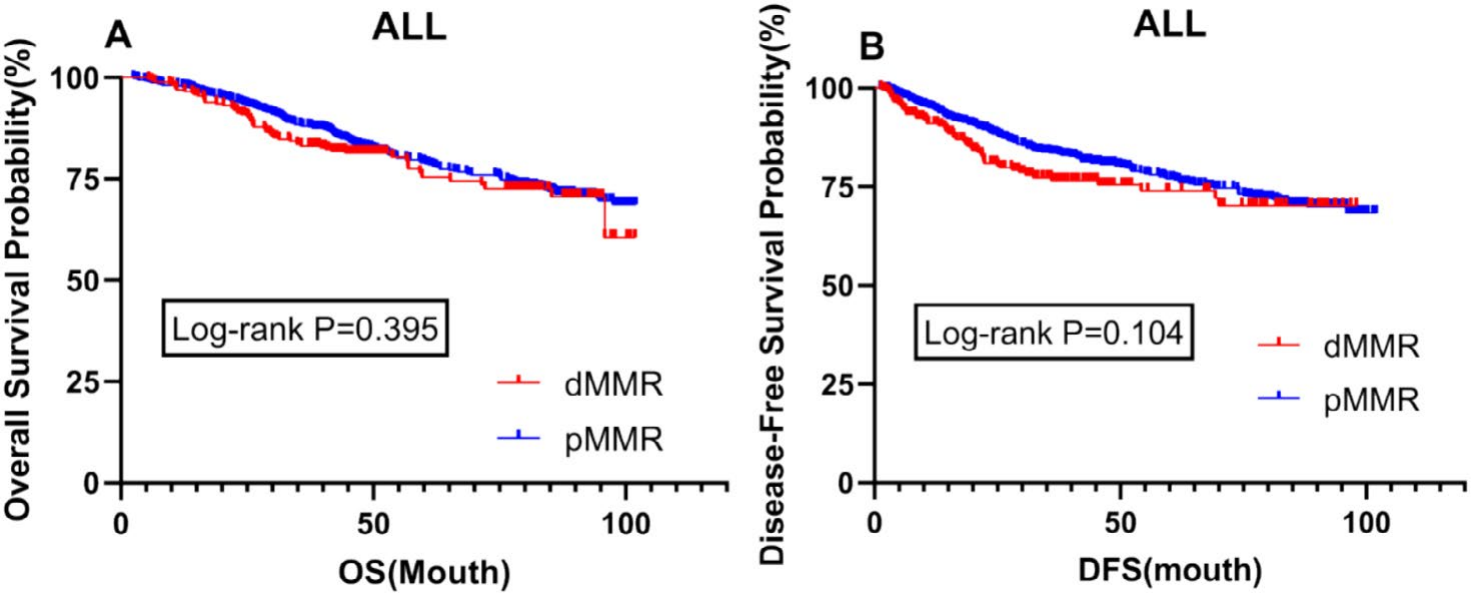
Survival analysis based on microsatellite status in the overall study population (Figure 2A). Kaplan‐Meier curves of overall survival (OS) for patients; (Figure 2B) Kaplan‐Meier curves of disease‐free survival (DFS) for patients. DFS, disease‐free survival; dMMR, mismatch repair deficiency; OS, overall survival; pMMR, protein mismatch repair deficiency.

For patients with pathological TNM stages II and III, there was no significant difference in prognosis between dMMR and pMMR patients, either in overall survival (OS) or disease‐free survival (DFS) (*p* = 0.395 vs. *p* = 0.104) (Figure 2).

### 3.3 Prognostic Analysis of Patients With Different LNR Values

**FIGURE 3:**
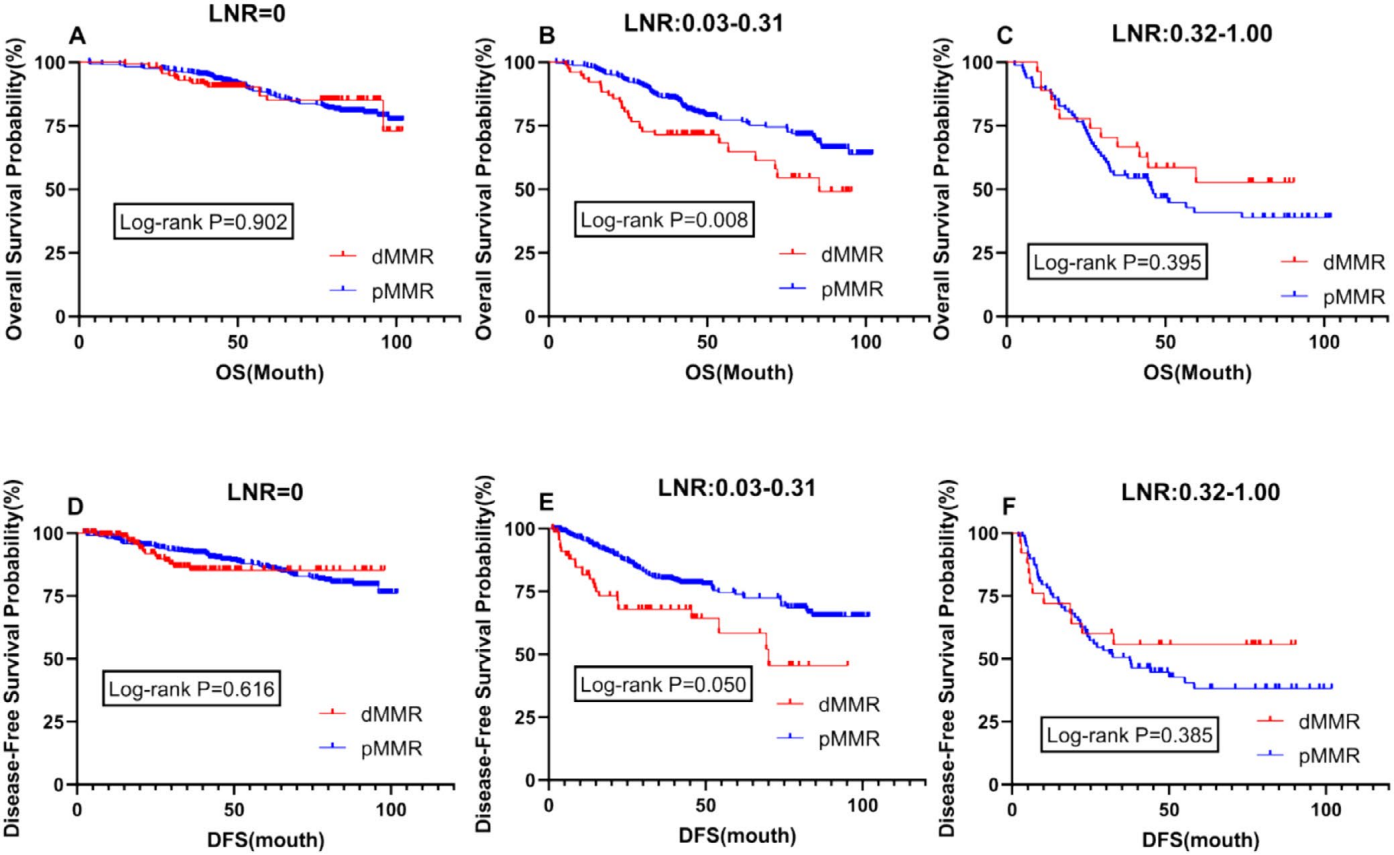
Stratified survival analysis based on different lymph node ratios in the overall study population. (Figure A and D) Kaplan‐Meier curve of overall survival (OS) and disease‐free survival (DFS) for patients with LNR=0; (Figure B and E) Kaplan‐Meier curve of overall survival (OS) and disease‐free survival (DFS) for patients with LNR ranging from 0.03 to 0.31; (Figure C and F) Kaplan‐Meier curve of overall survival (OS) and disease‐free survival (DFS) for patients with LNR ranging from 0.32 to 1.00 DFS, disease‐free survival; dMMR, mismatch repair deficiency; LNR, lymph node ratio; OS, overall survival; pMMR, protein mismatch repair deficiency.

Survival prognosis analysis was conducted for patients with different LNR values, revealing significant differences in both OS and DFS between dMMR and pMMR patients within the LNR range of 0.03–0.31 (*p* = 0.008 vs. *p* = 0.050) (Figure 3B,E). Additionally, further analysis indicated no difference in prognosis between the two patient groups at an LNR value of 0 (*p* = 0.902; 0.616) (Figure 3A,D). However, at higher LNR values ranging from 0.32 to 1.00, although not statistically significant, the prognosis for dMMR patients was better than that for pMMR patients, contrary to the prognosis within the LNR range of 0.03–0.31 (Figure 3C,F).

### 3.4 Impact of Postoperative Adjuvant Chemotherapy (ACT) on Prognosis in Patients With LNR (0.03–0.31)

**FIGURE 4:**
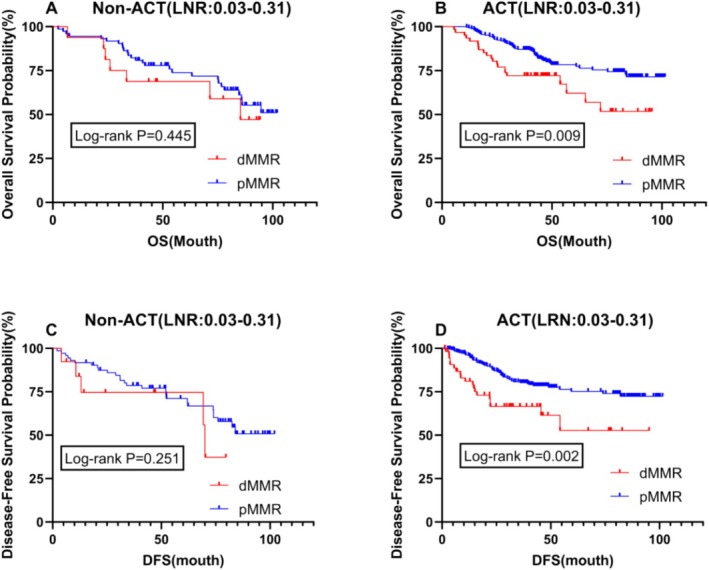
Survival analysis of different microsatellite status based on postoperative adjuvant chemotherapy. (Figure A and C) Kaplan‐Meier curve of overall survival (OS) and disease‐free survival (DFS) for patients with Non‐ACT (LNR:0.03‐0.31); (Figure B and D) Kaplan‐Meier curve of overall survival (OS) and disease‐free survival (DFS) for patients with ACT (LNR:0.03‐0.31) ACT, adjuvant chemotherapy; DFS, disease‐free survival; dMMR, mismatch repair deficiency; LNR, lymph node ratio; OS, overall survival; pMMR, protein mismatch repair deficiency.

In patients with stages II and III, prognostic analysis based on MMR status showed that postoperative adjuvant chemotherapy was a significant influencing factor. Therefore, we conducted a stratified analysis related to ACT for patients with an LNR between 0.03 and 0.31. The results revealed no difference in OS and DFS between dMMR and pMMR patients in the untreated group, with *p* values greater than 0.05 (Figure 4A,C). However, in the group receiving postoperative chemotherapy, there was a significant difference in prognosis for both OS and DFS (*p* = 0.009 vs. *p* = 0.002), with dMMR patients exhibiting a significantly worse prognosis than pMMR patients (Figure 4B,D). An LNR (Lymph Node Ratio) value between 0.03 and 0.31 may reflect a moderate degree of lymph node involvement, which significantly impacts prognosis. A lower LNR typically indicates fewer lymph node metastases and is associated with better survival rates, while a higher LNR implies more extensive metastasis, suggesting a poorer prognosis.

### 3.5 Impact of ACT on Prognosis in Patients With Different MMR Status in LNR (0.03–0.31)

**FIGURE 5:**
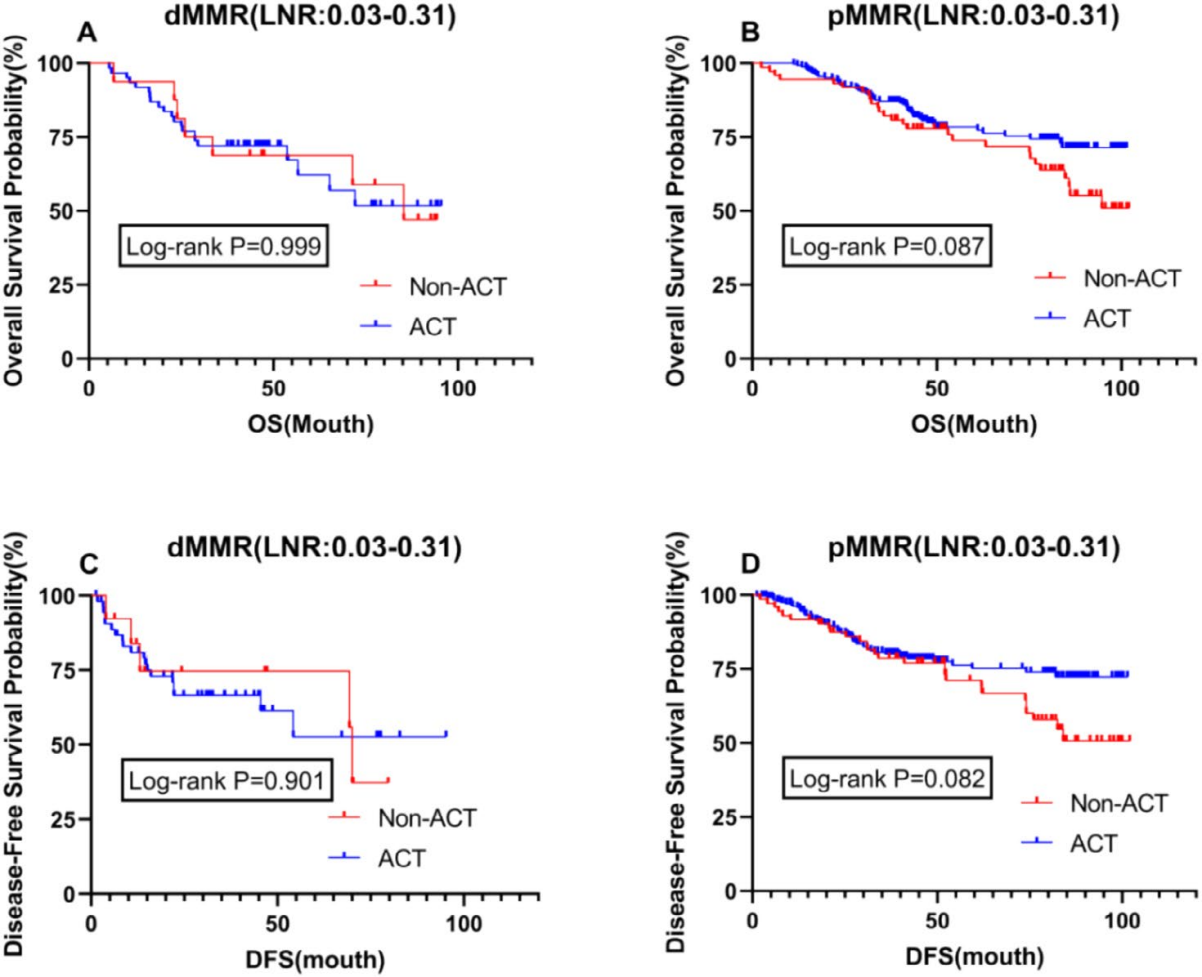
The impact of postoperative adjuvant chemotherapy on survival stratified by different microsatellite status. (Figure A and C) Kaplan‐Meier curve of overall survival (OS) and disease‐free survival (DFS) for patients with dMMR (LNR:0.03‐0.31); (Figure B and D) Kaplan‐Meier curve of overall survival (OS) and disease‐free survival (DFS) for patients with pMMR (LNR:0.03‐0.31). ACT, adjuvant chemotherapy; DFS, disease‐free survival; dMMR, mismatch repair deficiency; LNR, lymph node ratio; OS, overall survival; pMMR, protein mismatch repair deficiency.

To further validate the survival impact of postoperative adjuvant chemotherapy on patients with different MMR statuses, we conducted an intragroup stratified analysis for dMMR and pMMR patients. The results showed no difference in OS and DFS between dMMR patients, irrespective of whether they received postoperative adjuvant chemotherapy, with P values close to 1 (Figure 5A,C). However, among pMMR patients, those who received postoperative chemotherapy had a better prognosis than those who did not, although this difference was not significant (Figure 5B,D). The efficacy of ACT varies among patients with different LNR values. For patients with low LNR, especially those with dMMR, ACT may not significantly improve prognosis, as these patients generally have a better prognosis and are less likely to benefit from chemotherapy. In contrast, for patients with high LNR, particularly those with pMMR, due to the extensive lymph node involvement, ACT becomes particularly important.

### 3.6 Overall Impact of ACT and MMR Status on Survival in Stages II and III Patients

**FIGURE 6:**
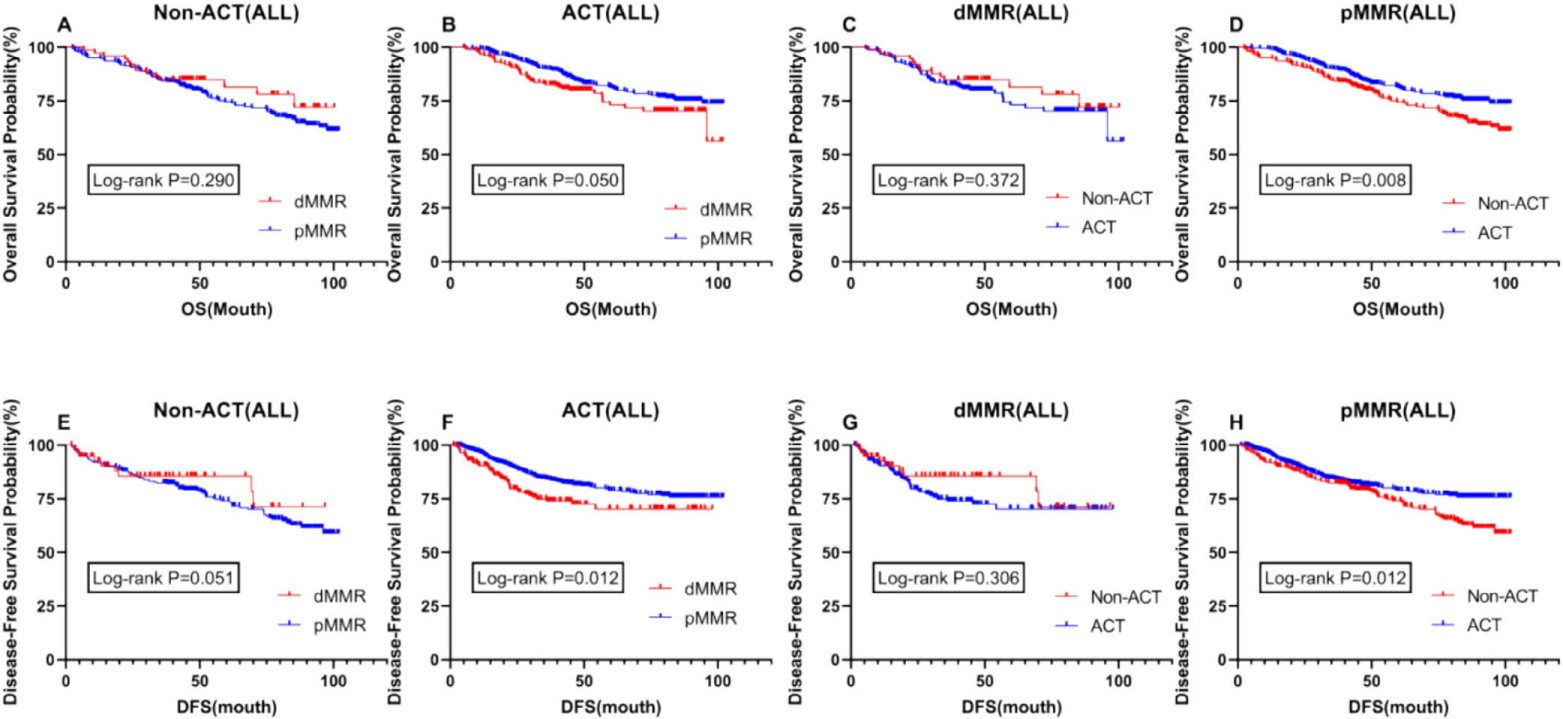
Stratified analysis of prognosis based on microsatellite status and postoperative chemotherapy in the general population. (Figure A and E) Kaplan‐Meier curve of overall survival (OS) and disease‐free survival (DFS) for patients with Non‐ACT(ALL); (Figure B and F) Kaplan‐Meier curve of overall survival (OS) and disease‐free survival (DFS) for patients with ACT(ALL); (Figure C and G) Kaplan‐Meier curve of overall survival (OS) and disease‐free survival (DFS) for patients with dMMR(ALL); (Figure D and H) Kaplan‐Meier curve of overall survival (OS) and disease‐free survival (DFS) for patients with pMMR(ALL) ACT, adjuvant chemotherapy; DFS, disease‐free survival; dMMR, mismatch repair deficiency; OS, overall survival; pMMR, protein mismatch repair deficiency.

To further refine our conclusions, we conducted a stratified analysis of all stage II and III patients, incorporating adjuvant chemotherapy (ACT) and MMR status for comparison. In the stratified analysis of the relationship between ACT and OS and DFS, we found results similar to those observed previously. Specifically, among patients with different MMR statuses, there was no difference in OS and DFS between dMMR patients with or without postoperative adjuvant chemotherapy. However, among pMMR patients, those receiving ACT had a significantly better prognosis in terms of both OS and DFS compared to those who did not receive ACT (*p* = 0.008 vs. *p* = 0.012) (Figure 6D,H). In the non‐ACT group, there was no significant difference in OS and DFS between dMMR and pMMR patients. However, in the ACT group, both OS and DFS were significantly prolonged in pMMR patients compared to dMMR patients (*p* = 0.050 vs. *p* = 0.012) (Figure 6B,F). Furthermore, among all patients, those with dMMR who did not receive postoperative adjuvant chemotherapy showed a better trend in OS and DFS compared to those who did receive ACT, although the difference was not statistically significant (Figure 6C,G). Additionally, in the non‐chemotherapy group, dMMR patients exhibited a better trend in prognosis compared to pMMR patients. However, this trend was reversed in the postoperative chemotherapy group, showing statistically significant results where dMMR patients had a significantly worse prognosis than pMMR patients (Figure 6A,E,B,F).

**FIGURE 7:**
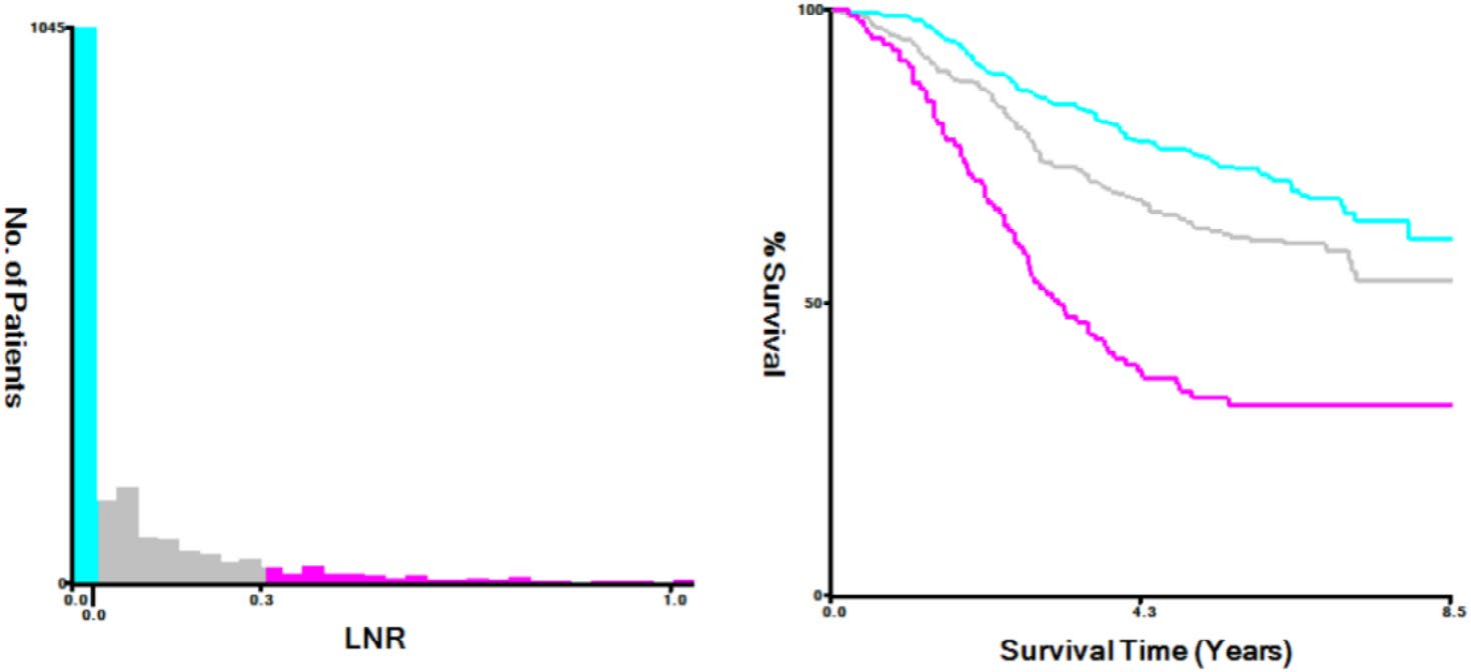
Selection of the LNR cut‐off value by X‐tile.

### 3.7 COX Multivariate Regression Analysis

Multivariate COX regression analysis identified factors such as age, T stage, Grade, LNR, and PNI as independent risk factors for OS, while age, T stage, Grade, and PNI were identified as independent risk factors for DFS. ACT was determined to be a favorable factor for DFS (Table 2).

**TABLE 2:** Multivariable cox survival regression analysis.

	OS		DFS	
	HR (95% CI)	*p*	HR (95% CI)	*p*
Sex	1.072 (0.823–1.396)	0.605	1.031 (0.790–1.347)	0.820
Age	1.396 (1.105–1.765)	0.005	1.340 (1.057–1.700)	0.016
T stage	1.709 (1.236–2.363)	0.001	1.591 (1.135–2.232)	0.007
N stage	1.348 (0.904–2.010)	0.142	1.391 (0.934–2.072)	0.104
Grade	1.535 (1.266–1.861)	< 0.001	1.581 (1.298–1.927)	< 0.001
LNR	1.343 (1.029–1.753)	0.030	1.284 (0.984–1.676)	0.065
Size	0.895 (0.687–1.165)	0.408	0.876 (0.669–1.148)	0.337
ACT	0.639 (0.484–0.843)	0.639	0.642 (0.483–0.853)	0.002
VNI	1.344 (0.972–1.859)	0.074	1.340 (0.963–1.865)	0.083
PNI	1.566 (1.107–2.217)	0.011	1.513 (1.061–2.157)	0.022
MMR	0.928 (0.683–1.262)	0.634	0.825 (0.593–1.146)	0.251

Abbreviations: ACT, adjuvant chemotherapy; dMMR, mismatch repair deficiency; LNR, lymph node ratio; LVI, lymphovascular invasion; MMR, microsatellite instability; pMMR, protein mismatch repair deficiency; PNI, perineural invasion.

## 4 Discussion

The role of microsatellite instability (MSI) in the prognosis and treatment guidance of colorectal cancer patients has attracted increasing attention among researchers, yet the specific impact of different MSI statuses, particularly dMMR, on the treatment outcomes of rectal adenocarcinoma patients remains unclear [11]. Data from the PETACC‐3 trial showed that tumor specimens characterized by MSI were more common in stage II disease than in stage III disease (22% vs. 12%, respectively; *p* < 0.0001), highlighting the clinical significance of studying MMR status in stage II and III rectal adenocarcinoma patients [29]. Additionally, the number of examined lymph nodes (ELN) has been confirmed to be associated with the prognosis of rectal cancer patients [30], but recent studies suggest that lymph node ratio (LNR) is superior to ELN in predicting prognosis, with some even indicating that LNR has higher prognostic value than MMR status [31, 32]. Therefore, our study analyzed the prognosis of stage II and III rectal cancer patients based on LNR and ACT, providing important clinical guidance for the treatment and prognosis of rectal cancer patients.

LNR, defined as the ratio of positive lymph nodes (PLN) to the total number of dissected lymph nodes (ELN) [28], has been identified as an important prognostic factor in various cancers, including lung and esophageal cancer [33, 34, 35]. The study by Ceelen et al. confirmed that in stage III colorectal cancer patients, the lymph node ratio (LNR) is an independent prognostic factor for overall survival (OS) [36], and the stratification significance of LNR is greater than that of traditional lymph node staging (N) [37]. However, the optimal cutoff value for LNR in rectal cancer patients remains unclear. Different studies report varying values for the lymph node ratio (LNR) due to disparities in patient demographics, surgical techniques, and the number of lymph nodes examined, which complicates the establishment of a uniform optimal cutoff point. In previous research, Li et al. reported cutoff values of 0.08, 0.25, and 0.50 [38], whereas in Zhang et al.'s study, the cutoff values ranged from 0.167 to 0.562 [25]. Consequently, various investigations have proposed distinct optimal cutoff values for LNR. Future large‐scale prospective studies, integrated with relevant molecular markers and further research tailored to specific patient populations, are warranted. Such efforts will enhance the precision of LNR as a prognostic tool and ensure more personalized treatment plans for colorectal cancer patients.

In our study, we examined LNR based on MMR status and combined MMR status with LNR to conduct prognostic analysis, further providing important clinical guidance for the prognosis of rectal cancer patients. Although overall prognosis did not significantly differ between dMMR and pMMR patients in our study, significant differences were observed based on LNR cutoff values of 0.03 and 0.31, providing clear prognostic reference indicators and ranges for clinical use.

Furthermore, our detailed stratified analysis based on MMR status, LNR, and ACT provided further guidance on prognosis and postoperative treatment for patients. We found that postoperative adjuvant chemotherapy (ACT) had a significantly detrimental effect on the prognosis of dMMR patients with stage II and III rectal cancer. This finding was consistent across analyses based on LNR values and stratification by ACT and MMR status.

Within certain ranges of LNR, there is no significant difference in prognosis between dMMR and pMMR patients. This may be due to the limited sample size, which reduces statistical power, as well as patient heterogeneity, such as variations in age, tumor location, and treatment methods. Furthermore, the strong prognostic impact of LNR may overshadow the effects of MMR status, especially in patients with higher LNR values where extensive lymph node involvement dominates the prognosis. Additionally, larger prospective studies are needed in the future, combined with relevant molecular markers, and further research targeting specific patient populations. This will help to improve the precision of LNR as a prognostic tool and ensure more personalized treatment plans for colorectal cancer patients.

Past studies have shown that rectal tumors with dMMR status exhibit different biological behaviors and clinical phenotypes in patients [6]. The impact of dMMR status on the treatment and prognosis of rectal cancer patients remains unclear [39]. Relevant research suggests that the loss of MMR proteins or MSI status may serve as a favorable prognostic marker for colorectal cancer patients [40]. These studies also indicate that II‐stage rectal cancer patients with dMMR status may not benefit from fluoropyrimidine‐based adjuvant chemotherapy and may even experience adverse effects [41], leading to a worse prognosis. However, the impact of dMMR is less pronounced in III‐stage rectal cancer patients and varies with tumor location in colorectal cancer [42, 43, 44]. However, Sargent et al. studied the prognosis between postoperative non‐chemotherapy and chemotherapy groups in colon cancer patients and concluded that the prognosis of the postoperative non‐chemotherapy group was better, contrary to previous studies [16]. This conclusion supports our results in rectal cancer patients. We conducted multiple stratified analyses, clearly demonstrating that in II and III‐stage rectal cancer patients, there was no difference in prognosis between the dMMR group and the non‐chemotherapy group, and the prognosis of dMMR patients after chemotherapy would be significantly worse, resulting in a significantly worse prognosis than pMMR. Conversely, in pMMR patients, the results are different. Studies by Cercek et al. and Ostwal V et al. also support our conclusion [45, 46].

However, regardless of whether based on LNR or ACT, MSI status detection has been strongly recommended as part of colorectal tumor evaluation [47]. Our study provides indicators for predicting prognosis and suggestions for postoperative treatment for patients with different MSI statuses. Our study provides clinical physicians with postoperative guidance that can be based on the patient's microsatellite status and the value of LNR. This study offers a cutoff value, but it may not be applicable to all situations. However, it still provides a direction.

However, it also has limitations. Firstly, the inherent recall bias in large retrospective studies is unavoidable. Additionally, excluding nearly half of the study population due to MMR status detection may reduce the credibility of our results. Furthermore, potential confounding factors were not balanced in survival analysis, necessitating larger, more comprehensive studies to confirm our conclusions. Nonetheless, our study is the first to analyze prognostic indicators based on MMR status and includes a large number of relevant patients.

## 5 Conclusions

In summary, our study analyzed the prognosis of II and III stage rectal cancer patients based on both microsatellite status and LNR. We found that LNR can serve as one of the clinical indicators for the prognosis of rectal cancer patients across different microsatellite statuses, providing reference cutoff values (0, 0.03, 0.31). Furthermore, our analysis of the impact of postoperative adjuvant chemotherapy (ACT) on prognosis concluded that it is advisable for II and III stage rectal cancer patients with dMMR to refrain from receiving any adjuvant chemotherapy postoperatively.

## Author Contributions

**Kailong Zhao:** conceptualization (equal), data curation (equal), formal analysis (equal), methodology (equal), software (equal), visualization (equal), writing – original draft (equal), writing – review and editing (equal). **Wenwen Pang:** data curation (equal), formal analysis (equal). **Xinyu Liu:** validation (equal). **Kemin Ni:** supervision (equal). **Weifeng Gao:** validation (equal). **Zhiquan Tan:** supervision (equal). **Jun Xue:** investigation (equal). **Weizheng Liang:** writing – review and editing (equal). **Xueliang Wu:** resources (equal). **Xipeng Zhang:** methodology (equal). **Xiaomin Su:** methodology (equal). **Chunze Zhang:** resources (equal).

## Ethics Statement

The study was approved by the Tianjin Union Medical Ethics Committee and carried out according to the Helsinki Declaration. Participants signed an informed consent form prior to the start of the study.

## Consent

The authors have nothing to report.

## Conflicts of Interest

The authors declare no conflicts of interest.

## Data Availability

Because of the limitations of the ethical validation of patient data and anonymity, the data set analyzed in this study is not available to the public but can be obtained from relevant authors upon reasonable request. If you are interested in acquiring data, please contact Professor Chunze Zhang.
